# Supplementary material for: Evaluate the stroke awareness of Palestinian undergraduate health students: A cross-sectional study on risk factors and warning signs
Source: PLoS One. 2025 May 22;20(5):e0324157. doi: 10.1371/journal.pone.0324157 (PMC12097636; doi:10.1371/journal.pone.0324157)
Supplement: Attachment — Submitted filename: PLOS_Review.doc [file pone.0324157.s002.doc]

Reviewer Comments:

Overall Comments: This is a good study regarding the assessment of knowledge and attitude of undergraduate students about stoke and its risk factors. However, there are some areas that require justifications and more detailed discussion to improve clarity in the manuscript.

Section_wise_Comments

1. **Abstract**

Comment 1: In methodology part, please mention and elaborate about the tool about. Also mention why among the other available tools this was chosen in the latter part of your manuscript.

Comment 2: It would be better if the readers would get to know the undergraduate levels and programs and their lengths in the introduction part and better to mention in the abstract.

Comment 3: In the conclusion part of your abstract, consider removing the last line as it seems out of place and consider adjusting somewhere more relevant.

2. **Introduction**

Comment 1: Ischemic stroke accounts for 87 percent…….cases? Where worldwide or only in Palastine, (add reference).

Comment 2: Consider rewriting line 75,76 and 77. It is difficult to comprehend.

Comment 3: Please add a map of the study site?

Comment 4: Line 108, the students were ….second year and beyond, I would like to know of which programs, and what is the length of these programs.

Comment 5: Is it not obvious that when the academic year progresses, knowledge regarding diseases increases. Please provide suitable explanations and citations to justify your independent variables?

Comment 6: If the sample size was calculated 385, why were 1006 responses collected, please explain.

3. **Study tool**

Comment 1: Please explain how the face validity, content validity of the tool was ensured?

Comment2 : Line 137 says, previous studies ,” what kind of studies are we talking about here, are these studies similar to this study? How?

Comment 3: line 169 “only one university, this contradicts with your statement in line 97 which says many universities.

4. **Results and discussion**

Comment 1: Why is academic score taken as an independent variable here, academic scores of which grade or which semester or which year?

Comment 2: line 332, what is the distinction between medical and non medical in your study?

Comment 3: Assessment of attitude has not been presented as an objective in your abstract and introduction but has been included in the manuscript, please consider revising your objectives.

Comment 4: Clearly outline the number of years that different programs run and the number of students that participated from each year from each of these programs for the ease of understanding the overall characteristics of the participants of your study.

Comment 5: “Other health sciences” what programs are under “other health sciences”? Clarify.

Comment 6: Please use an advance English language editing software to provide more clarity to your manuscript.
