# Supplementary material for: Evaluate the stroke awareness of Palestinian undergraduate health students: A cross-sectional study on risk factors and warning signs
Source: PLoS One. 2025 May 22;20(5):e0324157. doi: 10.1371/journal.pone.0324157 (PMC12097636; doi:10.1371/journal.pone.0324157)
Supplement: Attachment — Submitted filename: Response to Reviewers.docx [file pone.0324157.s003.docx]

**Dear editor and reviewer,**

**Reviewer #1**: This is a good study regarding the assessment of knowledge and attitude of undergraduate students about stoke and its risk factors. However, there are some areas that require justifications and more detailed discussion to improve clarity in the manuscript.

1. **Abstract**

Comment 1: In methodology part, please mention and elaborate about the tool about. Also mention why among the other available tools this was chosen in the latter part of your manuscript.

Response: Thanks for your comment. We couldn’t add more details in the abstract due to the word count limit. However, it was mentioned that the survey has been taken from a validated previous publications, and further details can be found in data collection tool section in the methodology.

Comment 2: It would be better if the readers would get to know the undergraduate levels and programs and their lengths in the introduction part and better to mention in the abstract.

Response: Thanks for your comment. (I have mentioned them in the methods-Study Population, inclusion, and exclusion criteria)

Comment 3: In the conclusion part of your abstract, consider removing the last line as it seems out of place and consider adjusting somewhere more relevant.

Response: The last line in the conclusion is deleted and proper modification is done.

2. **Introduction**

Comment 1: Ischemic stroke accounts for 87 percent…….cases? Where worldwide or only in Palastine, (add reference).

Response: Thanks for your comment. The percentage is for the percentage of ischemic stroke among global stroke cases. Proper referencing is added.

Comment 2: Consider rewriting line 75,76 and 77. It is difficult to comprehend.

Response: The lines are rewritten to be more comprehensive.

Comment 3: Please add a map of the study site?

Response: The map was inserted at the result section.

Comment 4: Line 108, the students were ….second year and beyond, I would like to know of which programs, and what is the length of these programs.

Response: Thanks for your comment. The participants of the study were separated into two categories: those studying the medical program which in turn separated into two phases: the first phase consists of students engaged in the preclinical study, while the second phase comprises students pursuing the clinical program in the medical field. In addition to medical students, undergraduate students pursuing other health-related studies including; nursing, pharmacy, dentistry, and allied health sciences.

The general undergraduate program length in Palestine ranges from a minimum of four years for nursing and allied health sciences programs such as medical imaging, medical analysis, and physiotherapy, five years for pharmacy and dentistry programs, and six years for the medicine program.

Details about the length of the study are added to the methods section

Comment 5: Is it not obvious that when the academic year progresses, knowledge regarding diseases increases. Please provide suitable explanations and citations to justify your independent variables?

Response: Thanks for your comment. The following paragraph has been added in the discussion section.

Notably, sixth year students in our sample scored the best in knowledge about scores. This is expected as they included only medical students in their final years as other specialties in the study do not have six year in the curriculum. The sixth year medical students are not only well-trained in terms of clinical practice but are also in the phase of preparation for exams which are both for graduation and international exams for residency purposes. This is in line with previous studies that supported the idea of better basic medical knowledge by students in their clinical years as they use it in diagnosis (10.1111/j.1365-2923.1988.tb00774.x., 10.1111/j.1365-2929.2004.02036.x.). The second best knowledge score was among fourth year students. This could be attributed to the fact that these students are near their graduate year among other health specialities. For medical students, it is the first clinical year and it comes after extensive exams for basic biomedical sciences as an obligatory requirement to transit to the clinical phase. So they still remember the information. The second year medical students scored the best among the basic year students as the courses of neuroanatomy, neurology and neurosciences are offered at the second year for medical and health sciences students in their curriculum A similar finding was reported in a previous publication concerning the better knowledge of students in genetics with the year in which they study the genetics course (10.1186/s12909-023-04996-6).

Comment 6: If the sample size was calculated 385, why were 1006 responses collected, please explain.

Response: Thanks for your comment. Even though the minimum calculated sample size based on a 95% confidence interval, and a 5% margin of error was 385, a total of 1006 responses were enrolled in the study to boost the analytical power of the study and to enhance the quality and reliability of the results.

**Study tool**

Comment 1: Please explain how the face validity, content validity of the tool was ensured?

Response: Thanks for your comment. The face validity was ensured by getting the questionnaire reviewed by specialists in stroke and experts in the field of health education. The content validity was ensured by doing modification on the tool after being taken from validated previous study in addition to conducting a pilot study to ensure understanding and suitability.

Comment 2: Line 137 says, previous studies ,” what kind of studies are we talking about here, are these studies similar to this study? How?

Response: Thanks for your comment. The referenced previous studies from which the questionnaire was adapted were about the knowledge of stroke including stroke risk factors and warning signs among undergraduate health students in Saudia Arabia.

Comment 3: line 169 “only one university, this contradicts with your statement in line 97 which says many universities.

Response: Thanks for your comment. The study sample included undergraduate health students studying at one of the universities located in the West Bank of Palestine.

Line 97: ‘’…undergraduate health students across many universities located in the West Bank of Palestine’’.

Line 169: ‘’….students studying medicine or health-related programs at one of the Palestinian universities’’.

3. **Results and discussion**

Comment 1: Why is academic score taken as an independent variable here, academic scores of which grade or which semester or which year?

Response: Thanks for your comment. The academic score is the cumulative academic score which reflects the student’s performance across multiple semesters or years, taking in consideration all the courses completed until the day of answering the survey. The sentence has been elaborated in method section.

Comment 2: line 332, what is the distinction between medical and non medical in your study?

Response: Thanks for your comment. The paragraph is rewritten to be more comprehensive since the meaning is the distinction between the students studying health programs other than medicine and the students studying medicine.

Comment 3: Assessment of attitude has not been presented as an objective in your abstract and introduction but has been included in the manuscript, please consider revising your objectives.

Response: Thanks for your comment. The objectives of the study has been elaborated at last paragraph of introduction as follows: “Therefore, this study aims to fill that gap by evaluating the knowledge of stroke risk factors and warning signs and attitudes toward stroke patients among healthcare undergraduate health students in Palestine.” In abstract, attitudes as objective has been added.

Comment 4: Clearly outline the number of years that different programs run and the number of students that participated from each year from each of these programs for the ease of understanding the overall characteristics of the participants of your study.

Response: Thanks for your comment. It is mentioned in the methods.

Comment 5: “Other health sciences” what programs are under “other health sciences”? Clarify.

Response: Proper description is added to the results: ‘’which is classified into three groups; preclinical medical students, clinical medical students, and other health sciences. The other health sciences group indicates the students studying health-related programs other than medicine, and include the doctor of dental program, doctor of pharmacy program, pharmacy, medical analysis, nursing, physiotherapy, anesthesia program, and hearing and speech programs as described previously in Table 1.’’

Comment 6: Please use an advance English language editing software to provide more clarity to your manuscript.

Response: Done.

**Reviewer #2:**

1. Unexpected higher awareness of stroke warning signs among preclinical students:
   The findings suggest that preclinical medicine students had higher awareness on recognizing stroke warning signs as compared to students who had clinical training, which contradicts expectations. Since clinical medicine students have more experience and direct patient exposure, they should logically perform better in recognizing stroke symptoms. The authors should explicitly address this unexpected result in the discussion section.

Response: Thanks for your comment. The following paragraph has been added in the discussion section.

“Even though the percentage of clinical phase-medicine students showed the lowest percentage of true answers regarding two of the risk factors which are excessive alcohol consumption and having migraines with aura, there were no significant differences between the preclinical and clinical medical students in achieving good knowledge about stroke based on the logistic regression. The lower percentage of true answers regarding alcohol consumption as a stroke risk factor could be attributed to the presence of studies stating that light-to-moderate alcohol consumption is associated with a decreased risk of ischemic stroke (<https://www.ahajournals.org/doi/full/10.1161/STROKEAHA.121.037590>, <https://www.mdpi.com/2308-3425/9/10/317>). Moreover, a previous study showed that alcohol consumption effect on stroke is affected by the genetic factor (<https://link.springer.com/article/10.1186/s12967-021-02904-4>).”

2. Potential response bias due to negative question framing: The question, "Do you think people who have a stroke cannot lead a happy life?" is negatively framed, which may have influenced responses. Negative framing can lead to acquiescence bias, where participants are more likely to agree even if they are unsure. It could also cause priming where participants focus on challenges rather than recovery possibilities, potentially overestimating negative perceptions. The authors could consider discussing this potential response bias under the limitations section.

Response: Thanks for your comment. Limitation acknowledged. Added to the limitation section.
