# Supplementary material for: Evaluate the stroke awareness of Palestinian undergraduate health students: A cross-sectional study on risk factors and warning signs
Source: PLoS One. 2025 May 22;20(5):e0324157. doi: 10.1371/journal.pone.0324157 (PMC12097636; doi:10.1371/journal.pone.0324157)
Supplement: Attachment — Submitted filename: Review_PLOS_AD.docx [file pone.0324157.s004.docx]

**Manuscript ID: PONE-D-24-57604R1**
Title: Evaluate the Stroke Awareness of Palestinian Undergraduate Health Students: A Cross-Sectional Study on Risk Factors and Warning Signs

**Overall Comments:** Thank you for submission of the revised version of your manuscript. It has improved a lot from its previous version. Still, many areas remain for improvement.

Please find section wise comments below:

**Abstract:**

1.The abstract starts with “A complete understanding” of..Im my view, understanding is a vague term and understanding something completely has no limits. Therefore, please use a suitable line to replace the statement, something that is more measurable.

1. This study aims to (you have written aimed to). Please consider writing in present tense as much as possible in the your abstract.
2. A survey “ questionnaire “, the word questionnaire is missing.
3. Please write how was attitute analysed or scored or what scale was used in your study.
4. The results in your abstract is too long. Please highlight major findings only in your abstract.

**Introduction:**

1. Line 64 what are the best practices? Please mention.
2. Please include your definition of medical and other undergraduates as operationalized in your study to introduce your study population.
3. Line 102, how many universities ?
4. Line 103, who developed the questionnaire, team work? You hav only mentioned from previous publications, please provide some context of those papers as well.
5. Line 107 .It can contribute…, please mention how to add more clarity.
6. Line 113..and beyond, please refrain from using such vague words. Please mention how many years in exact number.

**Methodology**

7. Please consider adding a table showing medical related streams and non medical related streams in a flowchart to give a comprehensive picture of how many were selected from which stream.
8. Why was 1006 samples used and probability sampling technique not applied if we has such a large sampling frame?
9. Please add the map after your study site section in the methodology part.
10. Again, please elaborate how the “attitude” variable was interpreted or only knowledge was interpreted, was it only presented descriptively?

**Discussion**

Please mention discussions regarding the attitude variable as well. Please add lines regarding how and in what ways attitude can be changed regarding stroke in Palestinian undergraduates. The discussion is very simple and needs to be significantly improved with strong recommendations for policy makers.

Overall: The manuscript lacks clarity at many parts. Please consider showing your work to a person fluent in English language.

Thank you . Best Regards
