# Supplementary material for: Evaluate the stroke awareness of Palestinian undergraduate health students: A cross-sectional study on risk factors and warning signs
Source: PLoS One. 2025 May 22;20(5):e0324157. doi: 10.1371/journal.pone.0324157 (PMC12097636; doi:10.1371/journal.pone.0324157)
Supplement: Attachment — Submitted filename: Response_to_Reviewers_auresp_3.docx [file pone.0324157.s006.docx]

**Dear editor and reviewer,**

**Thank you for your time and your valuable comments which we believe they helped us in improving the quality of our manuscript.**

**Kind regards,**

**Corresponding author**

6. Review Comments to the Author

Reviewer #1: Please improve your English language. Technically, the paper has improved so much from its last submission but the re are some language and grammar related issues can be improved to enhance the readability and clarity of your paper.

Response: thanks for your comment. The English language has been edited all over the manuscript as highlighted in red in the revised manuscript by authors and with the help of an English expert from our university to whom we add acknowledgment at the end of the manuscript.

Reviewer #2: The submission is well presented and acceptable for publication. All comments have been thoroughly addressed.

Response: thanks for your recommendation
